# It started off as a Cys, how did it end up like this? Identifying the extent of unmodelled oxidatively modified cysteines within the Protein Data Bank

**DOI:** 10.1107/S2059798326003943

**Published:** 2026-07-01

**Authors:** Samuel P. Foster, Anna J. Warren, C. Alistair Siebert, Jaswir Basran, Peter C. E. Moody

**Affiliations:** a Diamond Light Source Harwell Science and Innovation Campus DidcotOX11 0DE United Kingdom; b Department of Molecular and Cell Biology, Leicester Institute of Structural and Chemical Biology University of Leicester LeicesterLE1 9HN United Kingdom; University of Oxford, United Kingdom

**Keywords:** X-ray crystallography, macromolecular crystallography, radiation damage, Protein Data Bank, cysteine

## Abstract

Radiation damage to macromolecular structures remains a significant challenge for accurate structure solution by X-ray crystallography, leading to incorrect structural and chemical interpretation of the data. Site-specific radiation damage is insidious, typically unidentifiable solely from summary statistics, and is primarily discussed with reference to the predominant forms: disulfide-bond cleavage, metal-centre reduction and decarboxylation of acidic residues. A method is presented for identifying potentially oxidatively damaged cysteines by interrogating the accuracy of the built model within the electron density and the geometry of the difference density peaks surrounding a cysteine. We also highlight that cysteines located within protein active sites or that are in hydrolases are predisposed to this damage.

## Abstract 2

This work examines data contained within 118 215 structures in the Protein Data Bank for unmodelled difference density concomitant with unmodelled oxidatively damaged cysteines. This analysis demonstrates that site-specific oxidation of residues is a phenomenon of which the experimenter should be aware, and highlights the need for a suitable detection tool within refinement software.

## 1. Introduction

Whilst mitigation strategies for radiation damage to macromolecular structures exist, they are dependent upon the experimenter’s correct implementation during data collection, and do not completely eliminate damage, perhaps with the exception of XFEL sources (Williams *et al.*, 2025 ▸). Global radiation damage typically occurs at much greater doses (in units of grey, Gy, J kg^−1^) compared with specific radiation damage and is identifiable in reciprocal space by (i) a loss of high-resolution reflections which gradually progresses to lower resolution shells, (ii) an expansion of the unit-cell volume, (iii) a gradual increase in Wilson *B *factors, (iv) worsening scaling statistics and (v) often an increased mosaicity (Garman & Weik, 2017 ▸). Site-specific damage, conversely, often occurs at much lower doses and is only identifiable in real space. The development of X-ray-induced damage to a macromolecule has been widely studied, with various events identified, and the progression of damage is largely understood (Garman & Weik, 2023 ▸). Metal centres are reduced (<10 kGy; Ebrahim *et al.*, 2019 ▸), disulfide bonds are cleaved (∼200 kGy; Bhattacharyya *et al.*, 2020 ▸) and acidic residues are decarboxylated (∼4000 kGy; Fioravanti *et al.*, 2007 ▸). Other phenomena have also been reported, including loss of the methylthio group from methionine and selenomethionine residues (Holton, 2007 ▸), C—*X* bond cleavage in halogenated inhibitors (Rodrigues *et al.*, 2024 ▸) and photolysis of other heavy-atom derivatives, including bromo, iodo and mercurial derivatives (Garman, 2021 ▸).

Specific damage to structures is therefore a widespread problem within crystallography, leading to incorrect conclusions on structure–function relationships, inaccurate models for structure prediction or drug design, and unsuccessful experimental phasing (McCoy & Read, 2010 ▸; Taberman, 2018 ▸). To alleviate this issue, dose-aware data-collection strategies such as those employed on I04 at Diamond Light Source (Flaig *et al.*, 2025 ▸) and simulations using *BEST* (Bourenkov & Popov, 2010 ▸) or *RADDOSE*-3*D* (Dickerson *et al.*, 2024 ▸) can be used to reduce the absorbed dose. Furthermore, previously collected data can be examined for damage using *RABDAM* (Shelley *et al.*, 2018 ▸) in the absence of dose calculations to infer the extent of damage within a structure, using the *B*_Damage_ metric (Gerstel *et al.*, 2015 ▸), or against all structures using *B*_net_-percentile (Shelley *et al.*, 2018 ▸; Shelley & Garman, 2022 ▸). X-ray-induced photoreduction of a sample is not wholly deleterious, as careful experimental design presents opportunities for phasing (Ravelli *et al.*, 2005 ▸), inferring function (Colletier *et al.*, 2008 ▸; Taberman *et al.*, 2019 ▸; Chance *et al.*, 2020 ▸) or studying redox reaction mechanisms (Hersleth *et al.*, 2008 ▸; Ebrahim *et al.*, 2019 ▸; Pfanzagl *et al.*, 2020 ▸).

**Figure 1:**
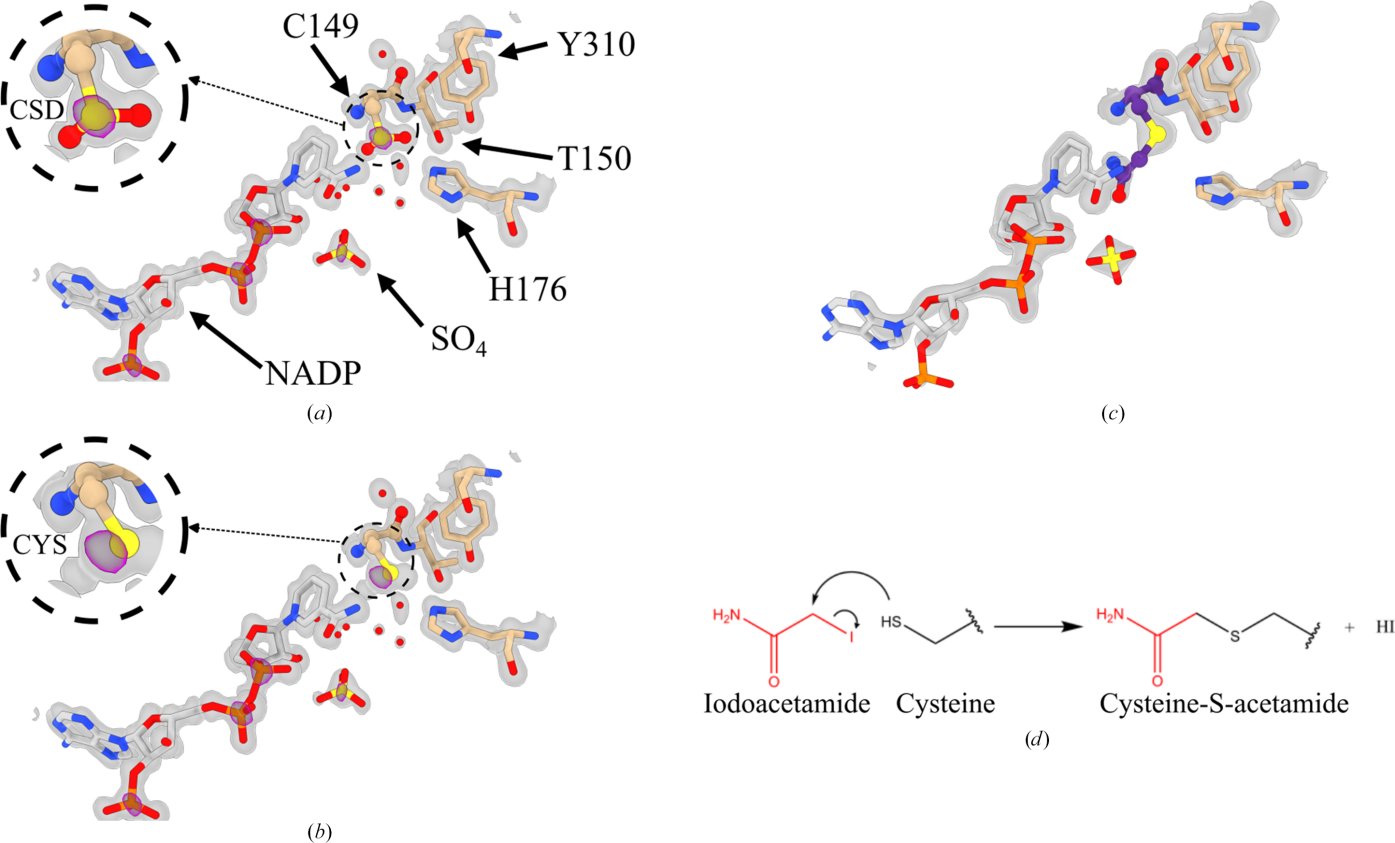
Structural basis of hpGAPDHA being nominally reduced. The best fit to the 2*mF*_o_ − *DF*_c_ (grey, contoured to 1.5σ) and anomalous difference (magenta, contoured to 5.0σ) maps is for a cysteine sulfinic acid (*a*) (cysteine sulfinic acid shown in the inset) and not the reduced sulfhydryl (*b*) (initially fitted cysteine in the inset). Crystals soaked in iodoacetamide dissolved; however, crystals produced with iodoacetamide-labelled hpGAPDHA show clear labelling of the active site (*c*) (carbamidomethylated cysteine presented in purple) and distal cysteines (not shown). As the S_N_2 reaction of cysteine with iodoacetamide requires cysteine to be reduced (*d*), successful labelling with iodoacetamide indicates that Cys149 is oxidized upon X-ray exposure. The PDB code for the NADP holoenzyme structure used in (*a*) and (*b*) is 9fq4; the iodoacetamide structure (Foster, 2025 ▸) (*c*) has not yet been deposited. This figure was prepared using *UCSF ChimeraX* v.1.10.1 (Pettersen *et al.*, 2021 ▸)

In a recent study of hpGAPDHA (Foster, 2025 ▸), one of the glyceraldehyde 3-phosphate dehydrogenases (GAPDHs) from the enteric human pathogen *Helicobacter pylori*, initial inspection of the *mF*_o_ − *DF*_c_ difference-density map at the active-site cysteine (Cys149) suggested that the cysteine existed in two conformations. Initial assignment of Cys149 existing in multiple conformations was made as the Harrigan and Trentham reaction scheme requires a reduced cysteine sulfhydryl (Harrigan & Trentham, 1973 ▸). The mechanism requires the reduced cysteine sulfhydryl (Fig. 1 ▸*b*) to attack the carbonyl of glyceraldehyde 3-phosphate, forming a hemithioacetal intermediate, which is subsequently oxidized to an acyl thioester intermediate. However, inspection of the anomalous difference density located P and S atoms, revealing that Cys149 was oxidized to a cysteine sulfinic acid [*R*SO(OH)] (Fig. 1 ▸*a*). As oxidation of hpGAPDHA would obviate catalytic activity, the protein was purified, assayed and crystallized in the presence of a reducing agent. Enzyme activity was observed in standard assays prior to crystallization and was abolished upon the addition of iodoacetamide, suggesting that the cysteine residue is nominally in a reduced state. Crystals were subsequently prepared using iodoacetamide-labelled hpGAPDHA, and the corresponding electron density clearly showed Cys149 as carbamidomethylated (Fig. 1 ▸*c*). Since Cys149 must be reduced for both the labelling reaction (Fig. 1 ▸*d*) and enzymatic activity, these results suggest that an X-ray-induced redox reaction is responsible for oxidizing Cys149 during data collection.

GAPDH is an enzyme with historical significance and an intensively studied reaction mechanism. Studies have shown that oxidation of the active-site cysteine regulates GAPDH function (Hildebrandt *et al.*, 2015 ▸), which is implicated in Alzheimer’s disease (Butterfield *et al.*, 2010 ▸). Searching the PDB for other GAPDHs [enzyme classification (EC) 1.2.1.12, 1.2.1.13, 1.2.1.9 and 1.2.1.59] with oxidized cysteines (PDB ligand codes CSD, CSX, OCS, CSU, CSO; Supplementary Table S2) yields 21 structures.^1^ Despite oxidized cysteines being modelled in the electron density, unless intentionally introduced with an oxidant this observation has been noted but not adequately explained (Boreiko *et al.*, 2021 ▸).

Cysteine oxidation caused by photochemistry has been described before and has been used to infer function (van den Bedem & Wilson, 2019 ▸). Similarly, the radiolytic generation of hydroxyl radicals from the bulk solvent by synchrotron radiation is utilized in hydroxyl-radical protein footprinting to infer the function of the protein of interest, identifying ligand interactions or high-order structural features (Xu & Chance, 2007 ▸; McKenzie-Coe *et al.*, 2022 ▸). However, given that this type of damage has not been widely reported within macromolecular crystallography, we suspect that this feature within electron-density maps is often ignored or incorrectly modelled. We have therefore sought to identify unmodelled oxidized cysteines within the PDB. Our workflow was initially trialled on all GAPDH structures before being applied to all of the X-ray-determined structures in the PDB. We estimate that 12.69% (∼15 000) of cysteine-containing structures exhibit this type of damage, and the prevalence is greater within thiol enzymes where the active-site cysteine is predisposed for reaction. Our work also shows that this damage does not correlate with *B*_net_-percentile, indicating that the mechanism of damage is independent of decarboxylation and suspected low-damage structures observe this phenomenon.

## 2. Methods

### 2.1. Dataset curation

For the preliminary analysis, an initial list of GAPDH PDB codes was produced to identify classification parameters. This list was generated by querying the RCSB PDB using the advanced websearch for (i) UniProt molecule name ‘glyceraldehyde-3-phosphate dehydrogenase’ or (ii) any enzyme classification number (EC) of 1.2.1.12, 1.2.1.13, 1.2.1.9 or 1.2.1.59 or (iii) an InterPro Lineage Identifier of IPR020830. This list was then filtered for structures solved by X-ray diffraction. This list was then used to download PDB-format coordinates and MTZ files from the *PDB-REDO* server (Joosten *et al.*, 2014 ▸). The total number of GAPDH records for the preliminary analysis was 225, corresponding to 2480 cysteines.

For the full-scale analysis of the PDB, the inclusion criteria were those entries which (i) were solved by X-ray diffraction, (ii) have experimental data, (iii) contain protein only, (iv) have a PDB-format coordinate file and (v) were released on or before 8 May 2025. Filtering the PDB-REDO repository for matching PDB codes gives 158 114 suitable structures for analysis and 1 209 111 cysteines.

To validate the performance of the classifier on the PDB, the whole PDB dataset was randomly subsampled, ensuring that none of the 225 GAPDH structures were included and the classifier labels were removed to eliminate bias. We elected to produce a dataset of 200 PDB entries, corresponding to 2137 cysteines. A dataset of this size was chosen to approximate the size of the initial GAPDH dataset and was deemed to possess statistical significance. The 2137 cysteines were manually inspected for oxidative damage and labelled as described for the initial curation of the GAPDH dataset.

For clarity, the datasets described are referred to as ‘GAPDH’, ‘whole PDB’ and ‘validation’, respectively. For all three datasets, *B*_net_-percentile was omitted post-calculation if the temperature data were either unavailable or greater than 120 K. This pruning was performed to ensure that comparing distributions of undamaged and oxidatively damaged cysteines is valid, as per the authors’ calculations of the metric (Shelley & Garman, 2022 ▸).

### 2.2. Data-processing workflow

For the preliminary analysis of GAPDH structures, each PDB entry had its map coefficients checked and fixed using *MTZFIX* (Agirre *et al.*, 2023 ▸; Winn *et al.*, 2011 ▸) prior to calculation of the 2*mF*_o_ − *DF*_c_ and 2(*mF*_o_ − *DF*_c_) electron-density maps using *FFT* (Agirre *et al.*, 2023 ▸; Winn *et al.*, 2011 ▸). Main-chain and side-chain atom statistics were then calculated using *EDSTATS* (Tickle, 2012 ▸). *EDSTATS* requires the use of legacy PDB-format coordinate files, hence explaining why CIF files were not used. Cysteine atoms were then extracted from the *EDSTATS* result before being passed into *Coot* (Emsley *et al.*, 2010 ▸) to find positive difference-density peaks of at least 3.0σ in the vicinity of the cysteine atoms. An output file was then written containing the number of peaks, the r.m.s.d. of the peaks, the distances in Å to the nearest cysteine S atom and the *XYZ* coordinates of the peaks. *RABDAM* (Shelley *et al.*, 2018 ▸) was then run for the structure to calculate an overall *B*_net_-percentile value and per-atom *B*_Damage_ value. As *B*_Damage_ is not directly comparable between structures, the mean *B*_Damage_ value for side-chain atoms is calculated per PDB entry and the sigma level for each cysteine side-chain *B*_Damage_ value is then calculated per structure. Finally, for every cysteine the following parameters are written to an output file for analysis: PDB ID, residue number, chain ID, residue RSZD, sulfur RSCC, number of peaks, minimum r.m.s.d. of those peaks, distance to closest peak, distance to furthest peak, *B*_net_-percentile, *B*_Damage_, σ(*B*_Damage_), data-collection temperature, high-resolution limit and data-collection source.

The workflow was broadly similar for the whole-PDB analysis; however, some optimizations were made to reduce the processing time with the ∼700-fold more structures. The PDB and MTZ files were distributed evenly over 24 jobs using a high-performance computing (HPC) machine. The longest part of the execution was the *RABDAM* calculation, which was run prior to the main execution loop for each structure and then merged into a final lookup table. Another lookup table is created containing data-collection temperature and enzyme classification number, which are not included within the PDB file header. These lookup tables are pre-loaded into memory to prevent multiple accesses of the same file from the HPC jobs. Each job then runs the main processing loop described above and produces a separate final output file, with a modified header (PDB ID, residue number, chain ID, residue RSZD, sulfur RSCC, number of peaks, minimum r.m.s.d. of those peaks, distance to closest peak, distance to furthest peak, *B*_net_-percentile, *B*_Damage_, σ(*B*_Damage_), temperature, EC number and oxidized cysteine prediction.

### 2.3. Initial identification of prediction parameters

Each cysteine in the GAPDH dataset was manually inspected and assigned as either undamaged or damaged with respect to cysteine oxidation. This was achieved by observing the electron-density maps and models generated by the data-processing pipeline in *Coot* (Emsley *et al.*, 2010 ▸), and then for cysteines with positive difference peaks in the vicinity, trying to either fit alternative conformations or fitting an oxidized cysteine into the density. Pairwise two-sample Kolmogorov–Smirnov (Massey, 1951 ▸) testing was calculated for ten variables which we predicted would identify damaged structures[residue RSZD, sulfur RSCC, number of peaks, minimum r.m.s.d. of those peaks, distance to closest peak, distance to furthest peak, *B*_net_-percentile, σ(*B*_Damage_), temperature and high-resolution limit]. After plotting the frequency distribution of each variable in both damaged categories, RSZD was selected as values larger than 3 correlated well with damaged structures. Spearman’s rank correlation coefficient (Spearman, 1904 ▸) was also calculated for each variable to identify those which correlated well with one another. Plotting the receiver operator curves for varying RSZD and distance thresholds then identifies which heuristics were most effective in identifying oxidatively damaged cysteines.

### 2.4. Evaluation of classifier performance with an unbiased test set

To evaluate the performance of the classifier, an unbiased test set was used to avoid potential overfitting used by evaluating against the GAPDH dataset. A 2 × 2 confusion matrix was created and performance metrics were calculated to identify the effectiveness of the prediction parameters. These metrics were then used to approximate the prevalence of oxidative damage at the cysteine and PDB-entry level using binomial aggregation.

### 2.5. Correlating active-site cysteines with predicted damage rates

Identification of EC numbers with active-site cysteines was determined using the M-CSA database (Ribeiro *et al.*, 2018 ▸), which provides annotated data of enzyme reaction mechanisms. A flat file of manually curated data from the database was downloaded, which contained a header: M-CSA ID, UniProt IDs, PDB (PDB ID of a representative structure for this mechanism), EC, residue/reactant/product/cofactor, PDB code (for the residue involved in the mechanism, for example ASP, CYS, HIS), chain/KEGG compound, resid/ChEBI ID, function location/name, role, role type and role group. The flat file was filtered for all cysteine residues in the ‘PDB code’ column and a set of all unique EC numbers was generated. The originally parsed data were then searched for each of these EC numbers to determine those which were predicted as damaged. Of the 837 unique EC numbers within the flat file, 172 were associated with an active-site cysteine. The odds ratio (OR) of being predicted with unmodelled oxidative damage was then calculated at the cysteine level and extrapolated to the PDB-entry level using the classifier performance metrics.

## 3. Results

### 3.1. *B*_net_-percentile does not correlate with radiation-induced cysteine oxidation

The *B*_net_-percentile metric was devised as a means of predicting the extent of radiation damage within a cryo-temperature protein structure solved by X-ray diffraction in the absence of dose information. *B*_net_ is calculated by computing the kernel density estimate of *B*_Damage_ values for aspartate and glutamate carboxyl O atoms and then taking the ratio of the distribution that lies either side of the median *B*_Damage_ value for all atoms. *B*_net_-percentile is then calculated by taking the percentile ranking of a structure’s *B*_net_ value with respect to the closest 1000 structures in a resolution range. The authors of the metric do not specify a value for either *B*_net_ or *B*_net_-percentile which means that a structure is damaged, but suggest that a *B*_net_ greater than 3.0 and/or a *B*_net_-percentile greater than 0.95 would warrant investigation (Shelley & Garman, 2022 ▸).

It is therefore appropriate to determine whether radiation-induced cysteine oxidation also correlates with *B*_net_-percentile, as well as other pertinent metadata. The high-resolution limit and data-collection temperature were taken from the PDB record for each structure assessed. The accuracy of the position of each cysteine within the surrounding electron density was calculated using the real-space difference-density *Z*-score (RSZD) and the real-space correlation coefficient (RSCC) metrics from *EDSTATS* (Tickle, 2012 ▸).

RSCC measures the agreement between ρ_obs_ and ρ_calc_ in the voxels surrounding the region of interest, *i.e.* side chain or whole residue, with 1 suggesting perfect agreement and 0 a model fitted to noise. The method used to determine the limiting radii of the voxels therefore directly alters the signal-to-noise ratio of the metric. Furthermore, the metric is reliant upon accurate scaling and atomic displacements, thereby evaluating both precision and accuracy in the metric. RSZD has been developed to deconvolute the model-to-map accuracy and precision and only assesses the model accuracy. RSZD indicates how significant the difference density Δρ in a region of the model is, expressed as a *Z*-score, with significant difference density present around regions with RSZD ≥ 3. RSZD is derived from the χ^2^ cumulative distribution function of Δρ for voxels surrounding the region of interest. This calculation normalizes Δρ based on the estimated uncertainty in difference density σ(Δρ) within the bulk solvent. The greatest cumulative probability over all subsets of voxels is then converted to the *Z*-score, after application of the Dunn–Šidák correction for multiple measures (Sokal & Rohlf, 1995 ▸). This correction is necessary because the greatest value is selected, meaning that with a greater number of voxels in the limiting radii there is a greater probability of a significantly larger difference density due to random chance, and the number of voxels in the limiting radii varies with the number of atoms per residue, the atom type and the isotropic *B* factor. RSZD was therefore selected to test for correlations with cysteine oxidation and model inaccuracies with respect to the local difference density, whereas RSCC was chosen to test for correlations in cysteine oxidation and both model inaccuracy and imprecision simultaneously.

The number of positive difference-map peaks, the minimum r.m.s.d. of the set and the distance range of the peaks to the cysteine SG atom were calculated using *Coot*. The number of map peaks was chosen as it should correlate directly with the oxidation status if they are well spatially separated. The minimum r.m.s.d. value of the difference density in the local vicinity is used as a proxy for the volume of the peaks, *i.e.* incorrect scaling that gives rise to many small peaks should not correlate as well as a single large peak, which is indicative of unmodelled atoms. The distance range for the peaks in the local vicinity was chosen as a potential means of discriminating between oxidatively damaged cysteines and other unmodelled atoms using prior chemical definitions. Finally, *RABDAM* was run for each structure to calculate *B*_net_-percentile and *B*_Damage_. For structures with no data-collection temperature metadata, or where the data-collection temperature was greater than 120 K, the calculated *B*_net_-percentile was omitted. As *B*_Damage_ is not comparable between structures, the sigma level of *B*_Damage_ for each cysteine side chain was calculated relative to the average *B*_Damage_ for the entire structure.

**Figure 2:**
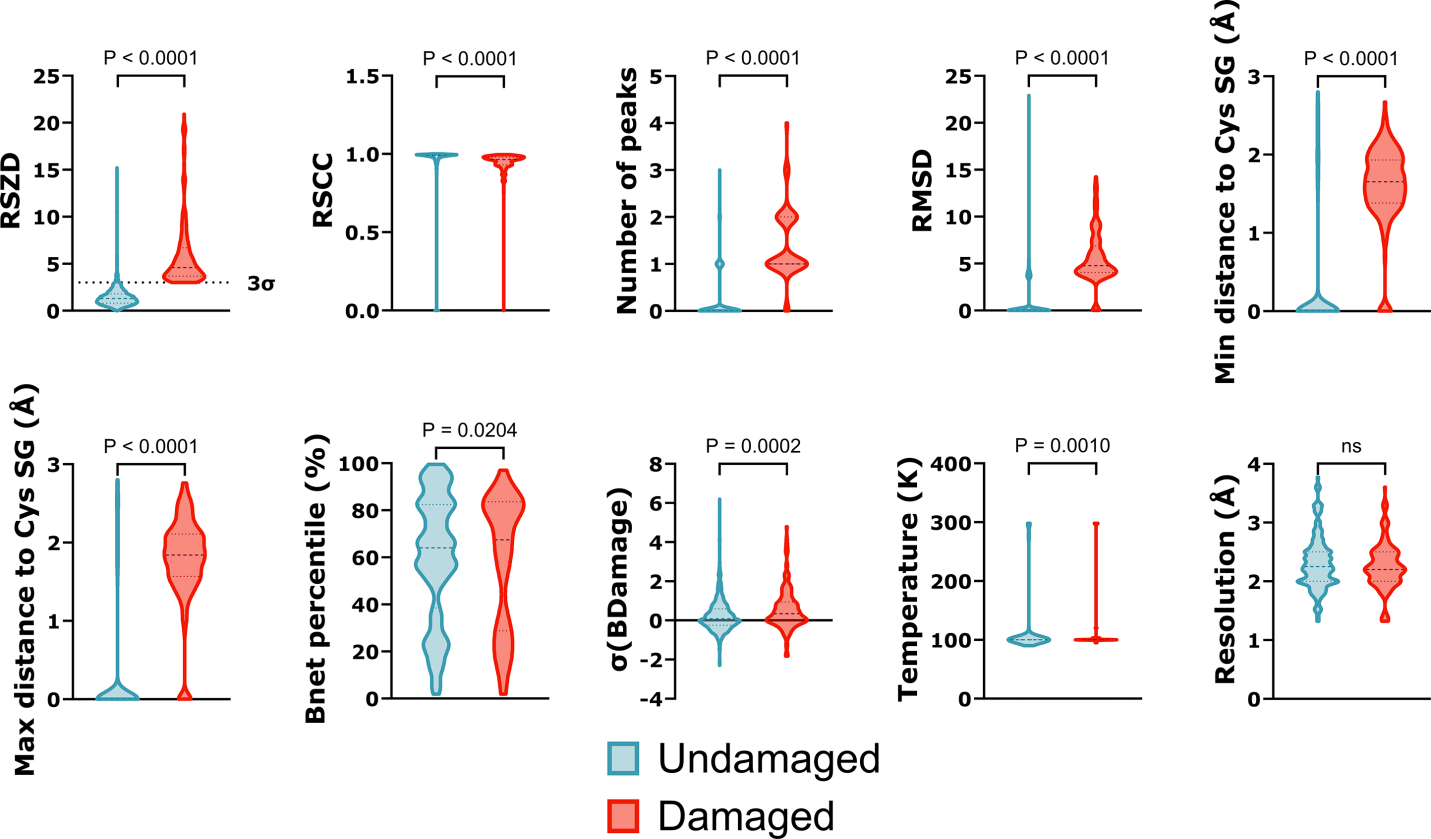
Violin plots comparing the distribution of undamaged (*n* = 2278) and damaged cysteines (*n* = 202) in the GAPDH dataset, depending on (top) RSZD, RSCC, number of peaks, minimum r.m.s.d. of local difference-density peaks and minimum distance of a difference-density peak to cysteine SG and (bottom) maximum distance of a difference-density peak to cysteine SG, *B*_net_-percentile (undamaged *n* = 2132, damaged *n* = 173), sigma level of the cysteine *B*_Damage_ relative to the rest of the structure (undamaged *n* = 2249, damaged *n* = 202), temperature (undamaged *n* = 2231, damaged *n* = 195) and resolution. 3.0σ is marked (dashed line) for RSZD as the suggested threshold by Tickle (2012 ▸). Evidently, although some distributions are statistically significantly different [RSCC, σ(*B*_Damage_) and temperature], their distributions do not lend themselves to setting threshold levels for damage prediction. The Kolmogorov–Smirnov *P*-value for each comparison is reported for each plot (unless otherwise stated *n* = 2480, ns = no significant difference), Dashed and dotted lines within each violin represent the median and quartiles, respectively. This figure was prepared using *GraphPad Prism* (version 10.6.0), GraphPad Software, Boston, Massachusetts, USA; https://www.graphpad.com.

To investigate possible correlations with the parsed metadata for each cysteine, pairwise two-sample Kolmogorov–Smirnov (KS, *n* = 2480) testing was performed for each parameter against oxidatively damaged (*n* = 202) and un­damaged (*n* = 2278) cysteines within the GAPDH dataset (Fig. 2 ▸). No significant differences in the distributions of resolution are observed depending on the cysteine oxidation status within the GAPDH dataset. However, significant differences (*P *< 0.0001) with large KS statistics are observed for RSZD, the number of peaks, r.m.s.d. and the distance between the cysteine SG atom and a difference-map peak (Supplementary Table S1). RSCC, *B*_net_-percentile, σ(*B*_Damage_) and temperature all have statistically significant differences in distribution; however, visual inspection of the distributions reveals that no meaningful thresholding could be applied. Similarly, except for RSCC, the KS statistic also suggests only small maximal differences in the distributions (Supplementary Table S1).

The two-tailed KS test was repeated for the full PDB dataset to test for differences in the distributions of *B*_net_-percentile in cysteines within datasets collected at cryo-temperatures, predicted with (*n* = 55 103) and without (*n* = 1 043 150) cysteine oxidation, to see whether the previous assumption made for the GAPDH dataset holds. The KS test indicated a statistically significant difference in the distributions of *B*_net_-percentile (*D* = 0.0142, *P* < 0.0001).

**Figure 3:**
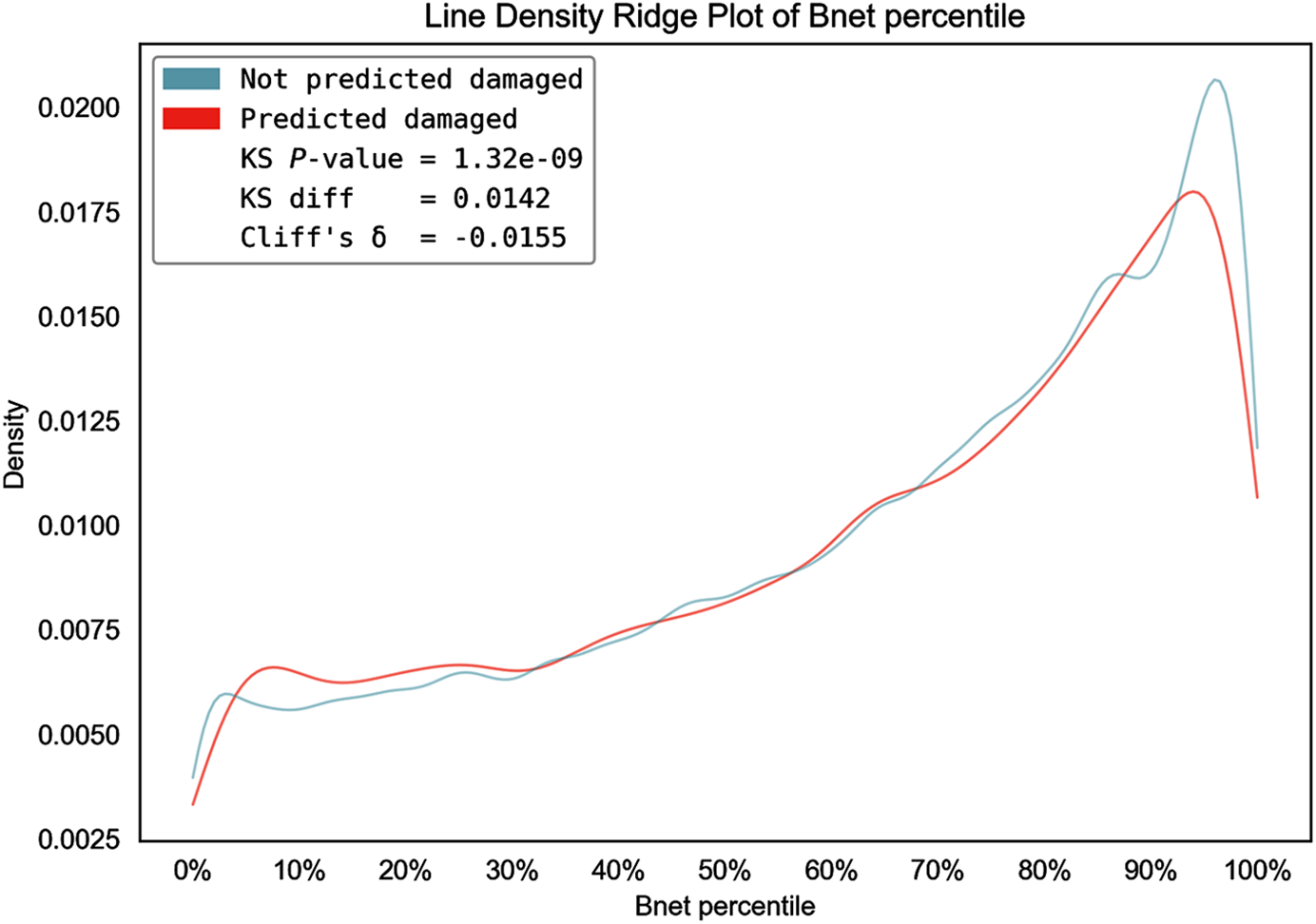
Kernel density estimate of *B*_net_-percentile values for structures predicted to (red) contain (*n* = 55 103) or (blue) not contain (*n* = 1 043 150) cysteines which are predicted as oxidatively damaged. Although there is a statistically significant difference in the distributions of *B*_net_-percentile, the distributions overlap considerably such that there is negligible effect size, as can be seen in the Cliff’s δ value. Therefore, *B*_net_-percentile cannot be accurately used to predict whether a structure contains an unmodelled oxidized cysteine.

Plotting the kernel density estimate of *B*_net_-percentile for the predicted damaged and undamaged structures (Fig. 3 ▸) shows that the two distributions are very similar. The bandwidth used for the kernel density was estimated using Silverman’s method (Silverman, 1986 ▸). We hypothesized that the significance was due to the large dataset size, which increases the statistical power, permitting the detection of small effects. Cliff’s δ was calculated as an effect-size measure for the two groups (Cliff, 1993 ▸). Although predicted non-oxidized cysteine-containing structures have marginally greater *B*_net_-percentile values, Cliff’s δ suggests a negligible effect size (δ = −0.0155) indicating limited practical significance in the difference of the distributions. Therefore, this validates the assumption that *B*_net_-percentile is not suited for identifying structures which potentially possess radiation-induced cysteine oxidation.

### 3.2. Predicting damage with real-space difference-density *Z*-score and distance to difference-map peak

**Figure 4:**
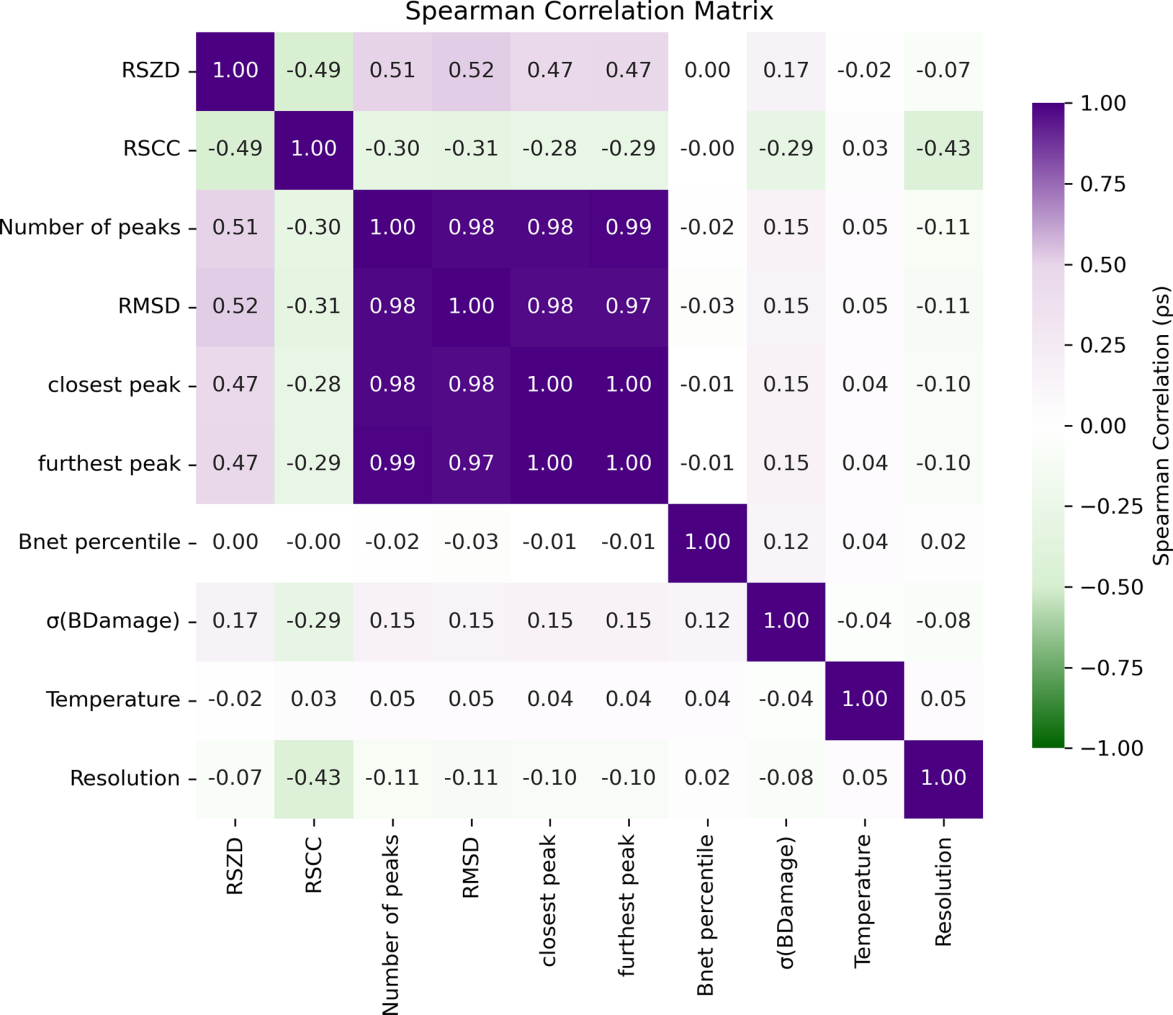
Spearman’s correlation matrix of the ten parameters tested for identifying oxidatively damaged cysteines in the GAPDH dataset. Parameters with a weak correlation (−0.2 < ρ_s_ < 0.2) are shown with desaturated colour. Almost perfect correlation exists between parameters which directly describe the positive difference density in the local vicinity of a cysteine, indicating that only one of these parameters need be used for the accurate prediction of oxidative damage.

To identify which parameters would be most effective for predicting whether a cysteine has unmodelled oxidative damage, Spearman’s rank correlation coefficient (ρ_s_, *n* = 2480) was calculated for the ten parameters previously identified for every cysteine present in the GAPDH dataset (Fig. 4 ▸). Assuming that weakly correlated parameters have a correlation coefficient −0.2 < ρ_s_ < 0.2, then *B*_net_-percentile and temperature are not correlated with any of the other parameters. A strong correlation exists between parameters directly related to the difference-density peaks surrounding a cysteine (the number of peaks, the minimum r.m.s.d. level of the associated peaks, the closest and furthest distance to a peak) and with RSZD. This reinforces the result that RSZD and a parameter associated with difference-map peak geometry would make good differentiators as they both describe identifiable features within the data for oxidative damage to cysteines. Conversely, RSCC is negatively correlated with parameters related to difference-density peaks, and inspecting the distributions for cysteines with and without oxidative damage in the GAPDH dataset (Fig. 2 ▸) shows there is not enough sensitivity for RSCC to be used as a classifier.

**Figure 5:**
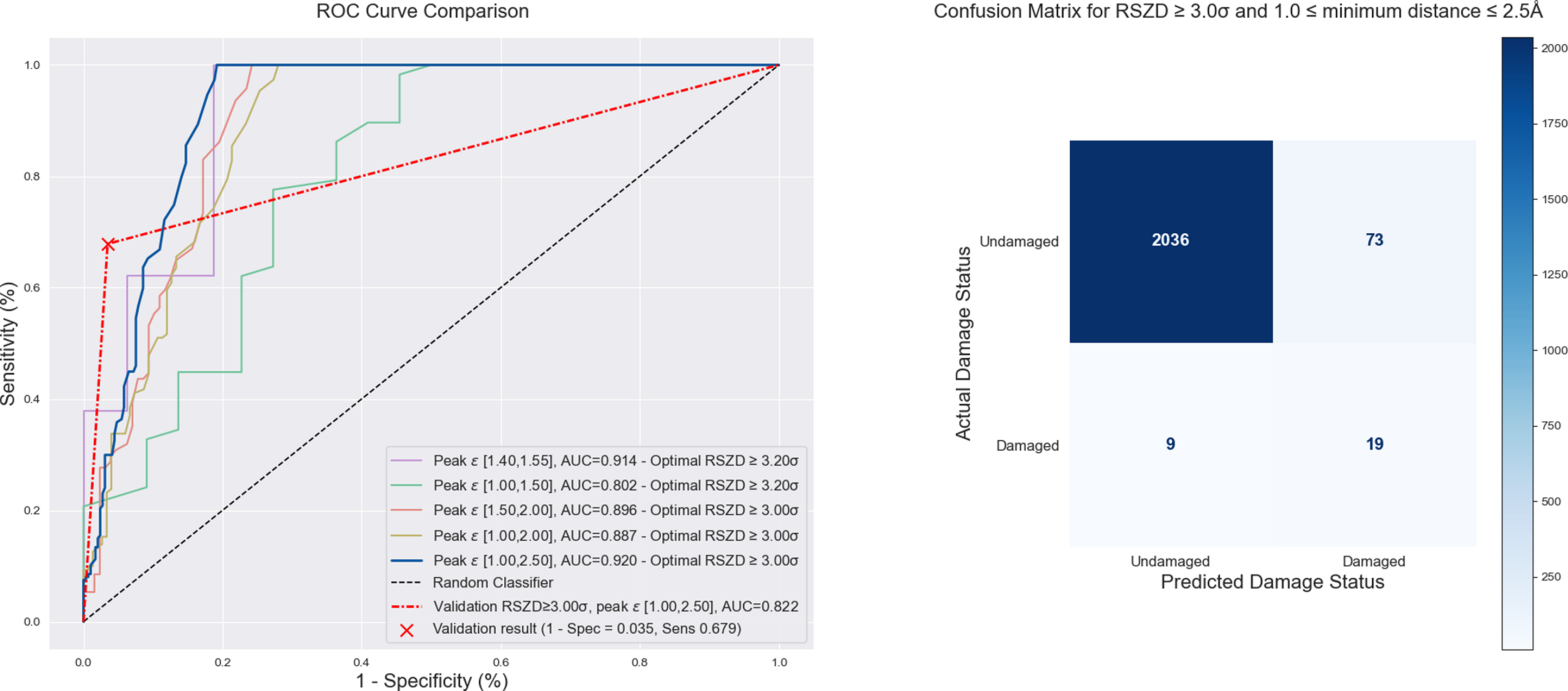
Left: receiver operator curves (ROC) for varying RSZD and distance to difference map peak for identifying potentially oxidatively damaged cysteines. Curves which are closer to the top-left hand corner, and therefore have a greater area under the curve (AUC), indicate better performance metrics. Each curve is plotted with a continually varying RSZD cutoff at a fixed distance threshold (1.40–1.55 Å, purple; 1.00–1.50 Å, green; 1.50–2.00 Å, orange; 1.00–2.00 Å, gold; 1.00–2.50 Å, dark blue). The RSZD threshold which maximizes Youden’s *J* statistic (Youden, 1950 ▸) is noted for each distance threshold. An RSZD of ≥3.0σ outperforms other cutoffs. Using chemically constrained values (1.40–1.55 Å) does not optimally select for potentially damaged sites compared with a 1.00–2.50 Å range. Therefore, the classifier used an RSZD ≥ 3.0σ and distance thresholds of 1.00–2.50 Å. The ROC curve of the unbiased validation dataset using the determined thresholds is shown as a red dashed line. Right: the confusion matrix for using RSZD ≥ 3.0σ and 1.00 ≤ distance to difference map peak ≤ 2.50 Å to classify the performance of the identification strategy is coloured by the number of cysteines within each class. The classification strategy correctly identifies 2036/2109 non-oxidatively damaged cysteines and 19/28 oxidatively damaged cysteines in the validation dataset. As there is a 75-fold difference in the classes, the Matthews correlation coefficient is the preferred test for robustness (Supplementary Table S3).

Within the ligand dictionaries available in the wwPDB (Berman *et al.*, 2003 ▸), the idealized S—O bond length of an oxidized cysteine is found to be approximately 1.4–1.5 Å (Supplementary Table S2). However, from the distribution of peak distances shown in Fig. 2 ▸, the peak distances lie between ∼1.0 and 2.5 Å in the manually inspected data. The idealized S—O bond lengths in the PDBe ligand dictionary do vary from other dictionaries such as those used in *Coot* (Emsley *et al.*, 2010 ▸) and observed in structures (our own structure PDB entry 9fq4 features bond lengths of 1.42 and 1.48 Å on the sulfinic acid). This discrepancy can exist for a host of reasons such as the p*K*_a_ of the local environment (Joosten *et al.*, 2022 ▸), resonance bonding within the side chain and different weights applied to ligand restraints or to the electron density (Priestle, 1994 ▸). Nonetheless, these factors do not fully account for the disparity with our observed distribution of peak distances, which could be attributed to a different bonding environment displacing the entire residue closer to the difference-map peaks or to errors in refinement for the unmodelled atoms. To select appropriate thresholds for identifying damaged cysteines, receiver operator curves (ROC) were plotted with differing thresholds of RSZD and the closest difference-map peak to the cysteine sulfur using the manually annotated GAPDH dataset (Fig. 5 ▸). Using RSZD ≥ 3.0σ and 1.00 ≤ closest distance to difference map peak ≤ 2.50 Å as a classification strategy was the optimal solution, with an area under the curve (AUC) for the ROC of 0.920. AUC is a nonparametric measure used for evaluation, where a value of 1.0 is for a perfect classifier and a random classifier has an AUC of 0.5.

Using the selected thresholds, the classifier was evaluated against a manually curated validation dataset (Supplementary Table S3). The AUC reduces to 0.822 when calculated for the unbiased validation dataset. The derived 2 × 2 confusion matrix for the classification strategy reveals that 2036/2109 undamaged cysteines and 19/28 damaged cysteines are correctly identified. A moderate correlation between the model’s predicted damage class and the actual damage class exists, indicated by a Matthews correlation coefficient (MCC) of 0.361. Due to the imbalanced classes with approximately 75 times the number of undamaged cysteines compared with damaged cysteines, MCC is advantageous for assessing the classifier robustness (Boughorbel *et al.*, 2017 ▸) compared with other performance metrics (Fig. 5 ▸, Supplementary Table S3). MCC is analogous to the Pearson correlation coefficient when used in binary classifications and is similarly bounded between −1 and 1, indicating perfectly anticorrelated or correlated, respectively, with 0 signalling a performance no better than random classification.

**Figure 6:**
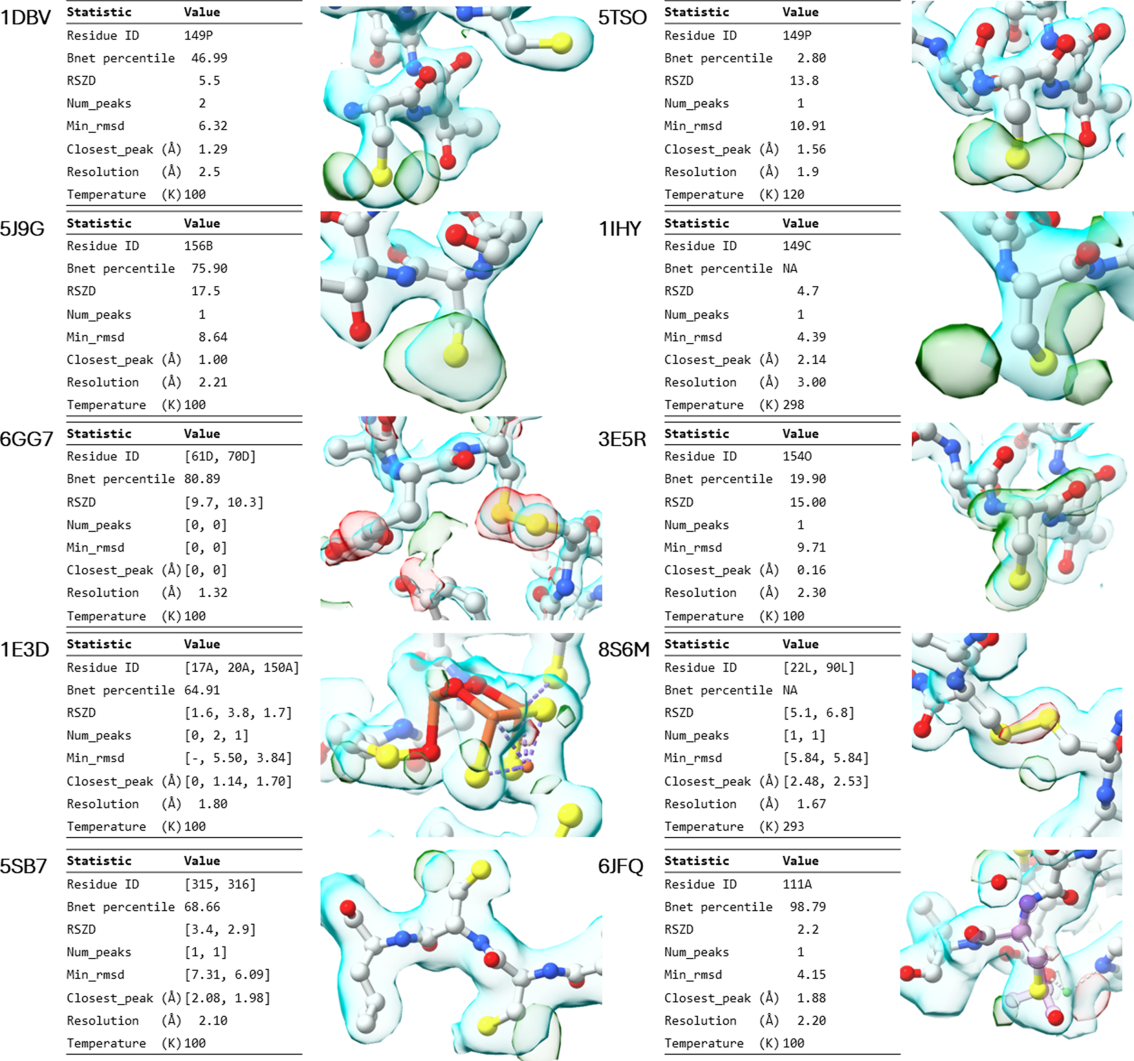
Metadata, models and density maps for representative examples of correctly classified cysteines, false positives and a false negative from the GAPDH dataset (PDB entries 1dbv, 5tso, 5j9g, 1ihy, 6gg7 and 3e5r) and validation dataset (PDB entries 1e3d, 8s6m, 5sb7 and 6jfq). PDB entry 6gg7 represents a true negative, where the cysteine is not predicted as oxidatively damaged despite the presence of clear reductive damage. For the false positives, PDB entries 3e5r, 1e3d, 8s6m and 5sb7, the reason for their flagging is depicted. PDB entry 6jfq represents a false negative where we were able to manually model a sulfinic acid into the electron density (purple) after deletion of the incorrectly placed water. There is no mention of radiation damage in structures which have a primary reference associated with them [PDB entries 1dbv (Didierjean *et al.*, 1997 ▸), 1ihy (Shen *et al.*, 2002 ▸), 6gg7 (McFarlane *et al.*, 2019 ▸), 8s6m (Rosen *et al.*, 2024 ▸), 1e3d (Matias *et al.*, 2001 ▸) and 5sb7 (Mühlethaler *et al.*, 2022 ▸)]. All of the crystals were produced in the presence of a reducing agent, with the exception of PDB entries 1e3d and 8s6m. Maps are contoured at 1.5 r.m.s.d. (2*mF*_o_ − *DF*_c_, blue) and ±3.0 r.m.s.d. (*F*_o_ − *DF*_c_, green/red).

Overall, this classification strategy is robust, as shown by the MCC and a diagnostic odds ratio of 58.9, and is highly effective at discriminating against non-oxidatively damaged cysteines, with a negative predictive value of 0.996. It is worth noting that the precision for the classifier (0.207) means that cysteines labelled as damaged have a 79.3% probability of not possessing oxidative damage. This can be attributed to the myriads of other reasons that can cause positive difference density to be present in the structure such, as unmodelled ligands, multiple conformations, errors in model building and poor scaling. Iron–sulfur clusters were noted to frequently possess positive difference density and would logically be sites prone to radiation damage, hence why they were frequently predicted as damaged (Fig. 6 ▸).

### 3.3. Confirmation of prediction parameters

To demonstrate the effectiveness of the filtering parameters in detecting oxidatively damaged cysteines, we manually inspected both the GAPDH dataset and the validation dataset after application of the identification strategy for examples of damaged cysteines (Fig. 6 ▸). PDB entry 1dbv (Didierjean *et al.*, 1997 ▸) is a typical result which shows the damage initially observed in the hpGAPDHA dataset, where active-site cysteines are oxidized preferentially over other peripheral cysteines. The damage is present in data collected both from synchrotron sources (for example, PDB entries 5tso, M. Dimova & Y. D. Devedjiev, unpublished work, and 5j9g, D. Patel, A. Pappachan & D. D. Singh, unpublished work) and from low flux-density sources such as sealed tubes (PDB entry 1ihy; Shen *et al.*, 2002 ▸) or rotating-anode generators (PDB entry 1dbv). At the time of writing, there are no structures of GAPDH determined using an XFEL deposited in the PDB. This supports the hypothesis that the damage is likely to be site-specific secondary damage, as the expected doses from the low flux-density instruments will be lower than those used in typical synchrotron-radiation datasets. Further evidence is provided from datasets which have low *B*_net_-percentile values (for example PDB entry 5tso, 2.797%), indicating that the unmodelled damage is present on the active-site cysteine only. The extent of oxidation cannot be estimated based on the number of peaks in the vicinity of the cysteine S atom, as demonstrated in PDB entry 5j9g, since a potentially triply oxidized sulfonic acid presents itself within the difference density as a single large peak and not three individual peaks. PDB entry 6gg7 (McFarlane *et al.*, 2019 ▸) further demonstrates why the distance thresholds are effective, as the positive difference density from a neighbouring decarboxylated glutamate, which is nonconcomitant with cysteine oxidation, does not cause the reduced disulfide bond to be flagged as oxidatively damaged.

However, false positives can occur due to similar cases of mismodeling closer to the cysteine being examined, such as in PDB entry 8s6m (Rosen *et al.*, 2024 ▸), where a photoreduced disulfide permits movement of the free thiol, resulting in unmodelled positive difference density. The reduction of a disulfide is a typical example of radiation damage within a structure, with negative density spanning the bond, and is well predicted with *B*_net_-percentile (if the dataset was collected at cryo-temperatures). PDB entry 8s6m was collected at room temperature, so *B*_net_-percentile should not be used for prediction of radiation damage. The higher crystal temperature probably permitted greater side-chain flexibility, enabling the free thiols to sample multiple occupancies, which is revealed by the positive electron density. Therefore, although disulfide-bond reduction is a misclassification by the model if strictly considering cysteine photo-oxidation, the inclusion of disulfides does enable the identification of these reduced sites, particularly within non-cryo-temperature datasets, and does permit some hypotheses to be drawn regarding the chemistry surrounding these sites. Other examples of mismodelling resulting in false positives include the cysteine being represented in the PDB file with low occupancy, as in the case of PDB entry 3e5r (Y. C. Tien, Y. H. Lin, S. L. Chang & C. J. Chen, unpublished work), positive difference density surrounding iron–sulfur clusters (for example, PDB entry 1e3d; Matias *et al.*, 2001 ▸) and alternate conformers not being modelled (for example, PDB entry 5sb7; Mühlethaler *et al.*, 2022 ▸). False negatives will also inevitably be present, particularly if the model has only partially been mismodelled. PDB entry 6jfq (I. H. Lee, T. H. Ho & L. W. Kang, unpublished work) exhibits a case where a cysteine was oxidized to sulfinic acid, but a water has been modelled in place of one of the sulfinate oxygens. The effect of this is likely to be a reduction in the *B*-factor of the thiol group, resulting in a low RSZD which falls below the threshold value.

In the structures presented here, where a publication is associated with the deposition, the crystals are reported as being produced in the presence of a reducing agent, except for PDB entries 1e3d and 8s6m, further supporting the occurrence of radiation-induced cysteine oxidation.

### 3.4. Expansion to the whole PDB

To predict the extent of unmodelled cysteine oxidation within the PDB, we applied this classification strategy to protein-only X-ray diffraction datasets, deposited with experimental data, prior to 8 May 2025. The PDB-REDO databank was then filtered using this list of structures, resulting in 158 114 structures suitable for analysis. We opted to use data deposited within the PDB-REDO databank so that the structures were each refined consistently with respect to one another. In total, 118 215 PDB entries were successfully processed, with the 39 899 not present accounting for those which do not have cysteines or have poor data (*i.e.* failed the processing). Our workflow suggests that of the 1 209 111 cysteines analysed, 4.94% are predicted to be oxidatively damaged. We are able to adjust this value to give an estimate of the true prevalence of oxidative damage to cysteines within the wwPDB, accounting for the performance metrics of our classifier. Given this apparent cysteine-level prevalence (AP) and the specificity (Sp) and sensitivity (Se) calculated with the validation dataset (Supplementary Table S3), we used the Rogan–Gladen correction (Rogan & Gladen, 1978 ▸) to estimate the true cysteine-level prevalence (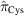) = 2.24%, 95% confidence interval (CI) [1.18%, 3.53%], 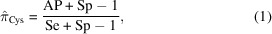
with 95% CI calculated by Lang and Reiczigel’s method for estimation given prevalence adjustment with a Rogan–Gladen correction (Lang & Reiczigel, 2014 ▸). Note that Se + Sp − 1 is Youden’s *J* statistic (Youden, 1950 ▸) utilized in the ROC plots in Fig. 5 ▸.

A cysteine flagged as oxidatively damaged therefore has an enhancement of being oxidatively damaged of ∼9.3-fold over a random cysteine (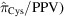. In the whole-PDB dataset, the number of cysteines per PDB entry follows a log-normal distribution, with a median of six cysteines per structure (*m*, data not shown). With an estimated 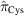 of 2.24%, and then using binomial aggregation (equation 2), we estimate the prevalence of PDB entries containing at least one unmodelled oxidatively damaged cysteine, 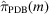, as 12.69%, corresponding to ∼15 000 structures. This assumes oxidatively damaged cysteines are randomly distributed across structures within the PDB, which we believe holds true for the number of structures present,
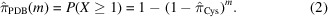
In our validation dataset 19/200 of PDB entries contained at least one unmodelled oxidatively damaged cysteine, yielding a prevalence of 9.50% with a Wilson’s 95% CI [6.17%, 14.36%] (Wilson, 1927 ▸), as shown in equation (3), which is compatible with our estimate of the prevalence across the whole PDB,
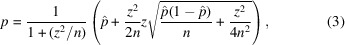
where *p* is the interval, *z* is the significance threshold (1.96 for 95%), 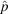 is the probability and *n* is the number of observations.

Furthermore, the prevalence within the validation dataset, π_Cys_ = 1.31%, Wilson’s 95% CI [0.91%, 1.89%], is congruent with our estimate of 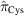 for the whole PDB, 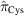 = 2.24%, Lang and Reiczigel’s 95% CI [1.18%, 3.53%].

### 3.5. Correlations with enzyme classification number

**Figure 7:**
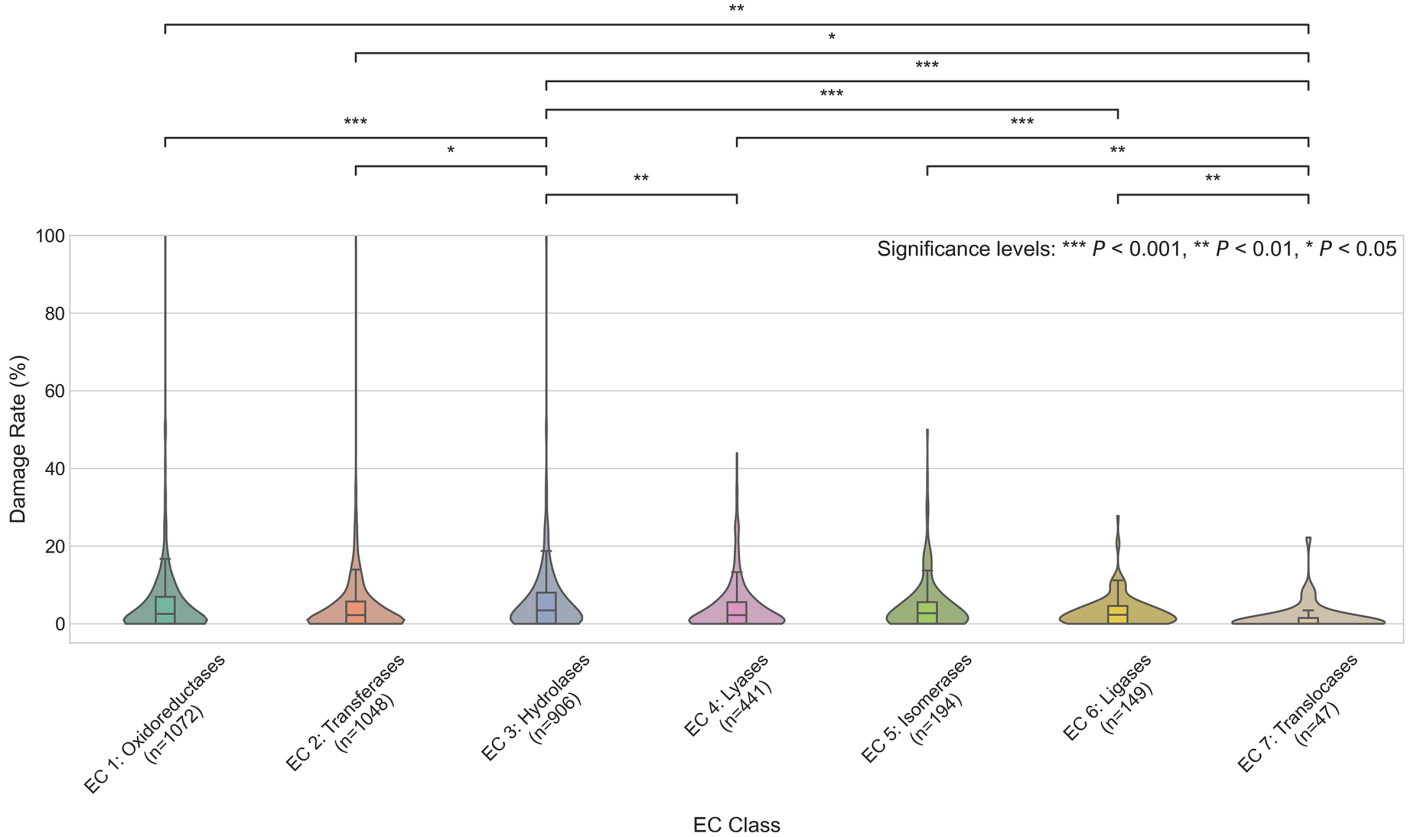
Violin plots of the predicted damage rates for cysteines in each EC class, annotated with the significance values calculated with Dunn’s test. *n* represents the number of unique EC classes per main EC class. The distribution of predicted cysteine-damage rates across EC numbers is not normal, thereby requiring nonparametric tests.

We aimed to determine whether cysteine residues which play a functional role in enzymes are more susceptible to this type of oxidative damage. We firstly calculated the proportion of cysteines which are labelled as damaged per unique EC number, then calculated the damage rates per EC class: EC 1 oxidoreductases, EC 2 transferases, EC 3 hydrolases, EC 4 lyases, EC 5 isomerases, EC 6 ligases and EC 7 translocases (Fig. 7 ▸). Prior to analysis, normality was assessed by visual inspection of the distribution of damage rates and the D’Agostino–Pearson test (D’Agostino & Pearson, 1973 ▸).

**Figure 8:**
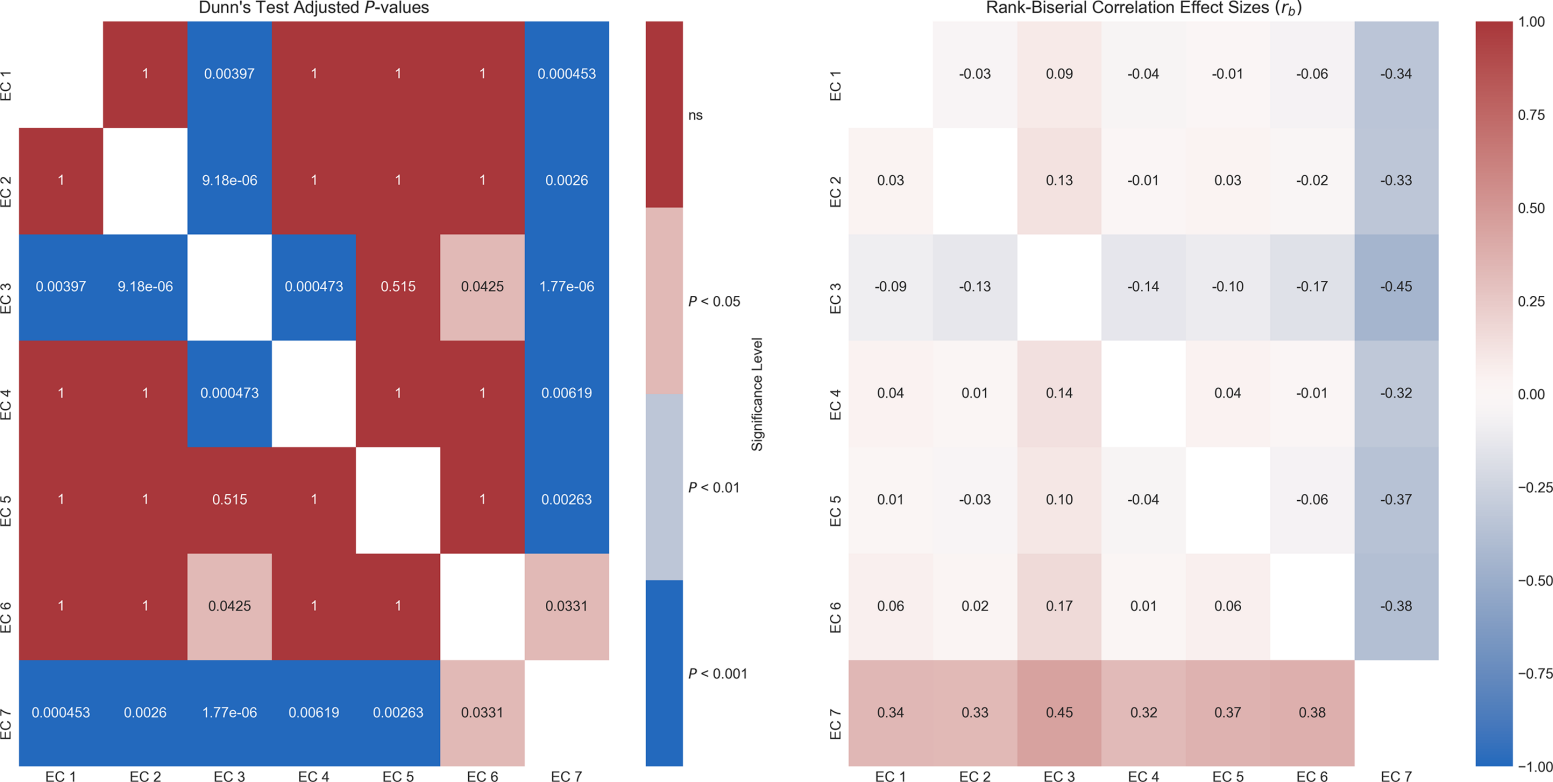
Results of Dunn’s post-hoc test for pairwise comparisons of damage rates across EC classes with (left) Bonferroni-adjusted *P*-values and (right) rank-biserial correlation effect sizes (*r*_b_). The magnitude of *r*_b_ can be interpreted as follows: trivial (*r*_b_ < 0.10), small (*r*_b_ = 0.10–0.29), moderate (*r*_b_ = 0.30–0.49) and large (*r*_b_ ≥ 0.50). The colour indicates whether a positive (red) or negative (blue) effect is observed between the groups’ damage rates when compared *X* versus *Y*; for example, EC 3 exhibits moderately greater damage rates compared with EC 7, *r*_b_ = 0.45.

As the data are not normal, nonparametric tests were used to compare damage rates across the seven EC classes. We use an α level of 0.05 for statistical significance testing. The Kruskal–Wallis *H*-test (Kruskal & Wallis, 1952 ▸) identified that there were significant differences in the damage rates of the seven enzyme classes [Kruskal–Wallis *H*-test *H*(6) = 52.6, *P* < 0.001]. Following the significant Kruskal–Wallis test result, pairwise comparisons were performed using Dunn’s post-hoc test with Bonferroni correction for multiple comparisons (Dunn, 1964 ▸). Dunn’s post-hoc test indicates ten statistically significant different pairings (Fig. 7 ▸). Rank-biserial correlation (*r*_b_) was also calculated per pairwise comparison, with the magnitude of *r*_b_ being a measure of the effect size and the sign indicating lower (negative values) or greater (positive values) observed damage rates (Fig. 8 ▸). The Dunn–Bonferroni test and *r*_b_ results show that translocase cysteines (EC 7) are significantly less likely to be predicted as oxidatively damaged compared with all other enzyme classes (*P* < 0.05, *r*_b_ = −0.32 to −0.45). In contrast, hydrolase cysteines (EC 3) are significantly more likely to be predicted as oxidatively damaged compared with five classes (*P* < 0.05, *r*_b_ = 0.09 to 0.45) except for isomerases (EC 5, *P* = 0.515). Interestingly, there are no additional significant differences for oxidoreductases (EC 1), despite this class being poised for redox chemistry.

We then sought to identify whether a structure which is annotated to possess an active-site cysteine is more likely to be predicted as damaged compared with those which are not. In the absence of residue-level active-site annotations, it is not possible to directly determine whether active-site cysteines are more likely to be predicted as oxidatively damaged compared with other cysteines. As a proxy, we identified EC numbers annotated to utilize active-site cysteines using the Mechanism and Catalytic Site Atlas (M-CSA; Ribeiro *et al.*, 2018 ▸), which provides annotated data of enzyme reaction mechanisms. The atlas was filtered for all cysteine residues, and a set of all unique EC numbers was generated. Of the 837 unique EC numbers in the atlas, 172 were associated with an active-site cysteine. Fisher’s exact test (Fisher, 1934 ▸) was used to determine whether there was a significant difference between cysteines which are linked to an EC number annotated in the M-CSA as utilizing a catalytic cysteine and those with any other EC number. Cysteines from PDB entries with EC numbers associated in the M-CSA with catalytic cysteines show greater predicted oxidatively damaged rates (5.39%) than cysteines from other EC numbers (4.26%) (Fisher’s exact *P* < 0.001; OR = 1.280, 95% CI [1.249, 1.313]).

We extended this analysis to the PDB-entry level, assigning a PDB entry as damaged if it contained ≥1 cysteines flagged as damaged. We then grouped the output depending on whether the PDB entry contained an EC number from the set of EC numbers annotated to utilize a catalytic cysteine (Supplementary Table S4) and then performed Fisher’s exact test to test for significance (Fisher, 1934 ▸). PDB entries which contain an EC number associated with catalytic cysteines are more likely to be predicted to contain ≥1 cysteines flagged as damaged (28.19% versus 25.16%, Fisher’s exact *P* < 0.001; OR ≃ 1.167, 95% CI [1.136, 1.198]). Because our classifier operates at the cysteine level, as PDB entries contain multiple cysteines, assigning a PDB entry as predicted to be damaged if it contains ≥1 cysteines means that the residue-level false-positive rate (FPR_res_; Supplementary Table S3) propagates to a large PDB-entry-level family-wise error rate [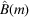],
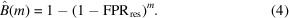
Furthermore, we can produce a conservative estimate for the expected sensitivity at the PDB-entry level [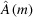] for detecting oxidized cysteines using equation (5),
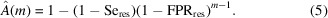
Thus, for a median of six cysteines per PDB entry, and using the residue-level FPR and sensitivity shown in Supplementary Table S3, 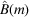 ≃ 19% and 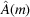 ≃ 73%. As a result, the predicted PDB-entry-level prevalence reported from the classifier is inflated to ∼26.32%, overestimating the estimated true prevalence 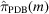 = 12.69%. Knowing this inflation in the prevalence, we use a Rogan–Gladen correction (Rogan & Gladen, 1978 ▸) to correct for the prevalence of PDB entries which contain ≥1 cysteines flagged as damaged in each group. Substituting specificity in equation (1) with
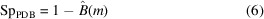
results in the point estimate of prevalence for PDB entries which contain ≥1 cysteines flagged as damaged,
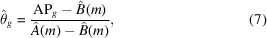
where 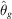 is the estimated prevalence for group *g* and AP_*g*_ is the apparent prevalence for group *g*. As 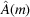 and 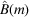 have been estimated by binomial aggregation for a median of six cysteines, we are unable to accurately calculate a Lang and Reiczigel interval. We therefore approximate the variance of the point estimate 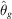 by dividing the variance of AP_*g*_ by the alternative form of Youden’s index (Youden, 1950 ▸) given in equation (7), weighted by the group size,
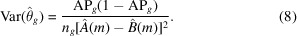
We then use the Delta method to estimate the 95% CI for the group prevalence (equation 9) and the individual odds for each group (equation 10),
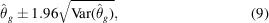

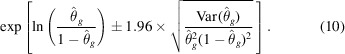


The 95% CI for the odds of each group is then used to calculate the 95% CI for the odds ratio using
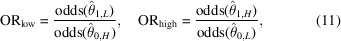
where 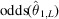 is the lower 95% interval for the odds of PDB entries with ≥1 cysteines flagged as damaged with an EC number within the catalytic cysteine set and 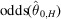 is the upper 95% interval for the odds of PDB entries with ≥1 cysteines flagged as damaged with any other EC number.

Application of the analytic correction indicates a higher true prevalence of unmodelled oxidative damage in PDB entries with catalytic cysteine-associated EC numbers (16.59%, Delta method 95% CI [15.82%, 17.36%]) compared with PDB entries with other EC numbers (11.00%, Delta method 95% CI [10.41%, 11.58%]), corresponding to a misclassification-adjusted odds ratio ≃ 1.61, 95% CI [1.43, 1.81].

## 4. Discussion

**Figure 9:**
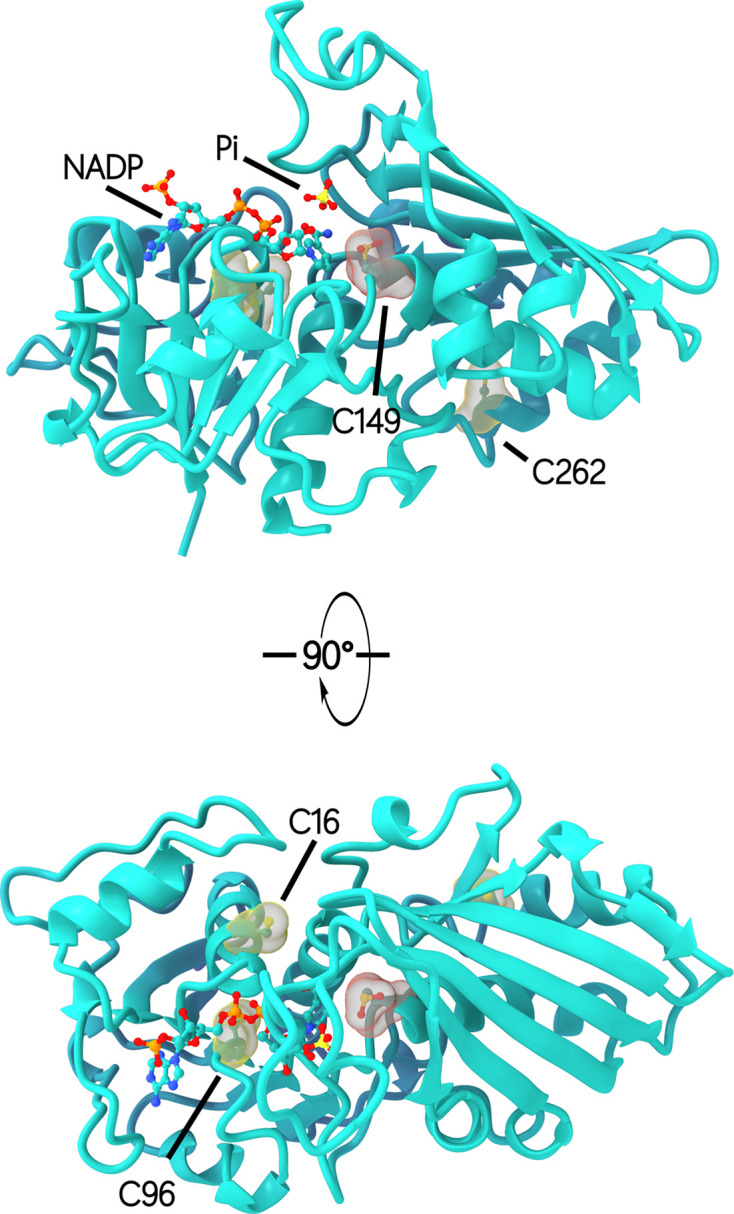
Preferential oxidation of active-site cysteines in GAPDHs. The active-site cysteine in hpGAPDHA (Cys149, red) is oxidized in the NADP holoenzyme structure, whereas the three other cysteines within the subunit (Cys16, Cys96 and Cys262, yellow) are not. The locations of the Pi site and NADP are also shown. This figure was created from PDB entry 9fq4 (Foster, 2025 ▸).

In this work, we have demonstrated that unmodelled cysteine oxidation within the PDB is widespread, potentially affecting 12.69% (∼15 000) of cysteine-containing structures, and is likely due to photo-oxidation. We employed heuristic checks to predict whether a cysteine is oxidatively damaged by interrogating the accuracy of the model within the electron density (RSZD) and the local geometry of positive difference-density peaks relative to the placed coordinates of the cysteine residue. The parameters used to define the heuristics were chosen based on calculated Spearman’s rank correlation coefficients between the ten parameters suspected to indicate damage outcome (Fig. 4 ▸). Four parameters (number of peaks, r.m.s.d. and closest/furthest difference density peak to the cysteine sulfur) correlated perfectly with one another, which is unsurprising as they are reporting the same features within the dataset; therefore, only one was required as part of the classifier. RSZD is strongly positively correlated with these parameters, which makes logical sense as RSZD is a measure of the difference density surrounding a residue, indicating the accuracy with which the model describes the electron density. RSZD and minimum distance to cysteine sulfur were selected to define the classifier due to their strong correlation (ρ_s_ = 0.47) and the clear differences in their distribution depending on damage outcome (Fig. 2 ▸). These heuristics were initially tested with a subset of the PDB containing solely GAPDH structures (Fig. 5 ▸) and readily identified cysteines with data suggesting unmodelled oxidative damage (Fig. 6 ▸). GAPDH structures were selected for preliminary investigation as we had observed cysteine photo-oxidation occurring only at the active-site cysteine and not distally within the structure, indicating a functional dependence on the predisposition to oxidative modification (Fig. 9 ▸).

The GAPDH dataset has a cysteine-level prevalence of oxidative damage of 8.15% (202/2480), Wilson’s 95% CI [5.23, 12.47], and within that dataset there is a PDB-entry-level prevalence of 32.5%, Wilson’s 95% CI [30.68, 34.37] (*i.e.* 32.5% of the dataset’s 225 PDB structures contain at least one damaged cysteine). If used on the full PDB dataset, it would overestimate the prevalence of oxidative damage as the model would be overfitted to the increased prevalence. The dataset was useful for initial setting of parameters of the classifier as the features were more common, which was then evaluated on the validation dataset constructed by randomly subsampling the full PDB dataset. Performance metrics indicate that the classifier is robust with an MCC of 0.361 and a diagnostic odds ratio of 58.9. The classifier performs especially well in discriminating against non-oxidatively damaged cysteines, with a 0.4% probability of a cysteine falsely identified as not oxidatively damaged [1 − negative predictive value (NPV), Supplementary Table S3]. The success in unmodelled damage being detected by the classification can be attributed to the well defined positive difference-map peaks which are readily identifiable by per-residue statistics and filtering these further based on proximity to the cysteine S atom. The heuristic employed does not utilize the expected bond lengths of oxidized cysteines as defined within their respective ligand CIF files (Supplementary Table S2), as this decreased the performance of the classifier. This could be attributed to displacements in the protein backbone or altered bonding patterns of the side chain within the solvent upon oxidation. Although there are a considerable number of false positives (PPV = 20.7%), these are largely attributed to other modelling errors (such as thiol-group displacement after disulfide-bond reduction, missing alternative locations or positive density surrounding electron-rich centres such as iron–sulfur clusters), which, whilst strictly decreasing the performance of the classifier, is still a useful endeavour to detect in order to improve the model. A multi-class classifier which could detect these known sources of error would have a greatly enhanced performance over this approach.

Radiation damage to macromolecular structures is typically discussed in terms of photoreduction events caused by the photoelectric effect, leading to either direct ionization of the macromolecule or reduction by the cascade of electrons generated in secondary ionization events (Taberman, 2018 ▸; Garman & Weik, 2023 ▸; Shelley & Garman, 2024 ▸). The suspected mechanism of photo-oxidation has been described previously, where radiolysis of the bulk solvent leads to reactive oxygen species (ROS), which react with residues in the local vicinity (George *et al.*, 2012 ▸; van den Bedem & Wilson, 2019 ▸). This phenomenon is also exploited in hydroxyl-radical protein footprinting (Xu & Chance, 2007 ▸; McKenzie-Coe *et al.*, 2022 ▸). In the absence of dose information, *B*_net_-percentile has been developed to estimate the degree of radiation damage expected within a structure by analysing the decarboxylation of aspartate and glutamate residues, as many proteins do not possess disulfides (Shelley & Garman, 2022 ▸). Decarboxylation of acidic residues is hypothesized to occur via the migration of electron holes along the protein backbone, which are subsequently filled by donation of an electron from acidic residues (Ravelli & McSweeney, 2000 ▸). Therefore, despite *B*_net_-percentile predicting damage based on an oxidative event, it does not accurately predict whether a system has undergone X-ray-induced photo-oxidation of a cysteine, likely due to the different mechanism by which residues are decarboxylated versus oxidized by the radio­lytically generated ROS.

The work presented here does not attempt to investigate the mechanism behind the photo-oxidation nor correlate with the local environment of the cysteines. This was explored in the GAPDH preliminary analysis, but the number of unique atoms in the local vicinity of cysteines across the relatively small dataset was large, resulting in low sample sizes per unique atom per damage class, meaning statistically insignificant correlations (not shown). Furthermore, expansion to the whole PDB using the approaches discussed in this work would likely become intractable and remain statistically insignificant, due to the wider variety of ligands. Perhaps a deep-learning approach could deconvolute the data and further enhance predictions of the oxidation state based on the local environment. We do note that when involved in an enzyme reaction, cysteine residues are expected to have a lower p*K*_a_ than those which are not catalytically active. Therefore, they would be ionized to a more reactive thiolate anion. This drove our hypothesis that catalytic cysteine residues would have a greater prevalence of photo-oxidation than noncatalytic cysteines.

As a proxy to directly analysing the local environment of every cysteine within the PDB, the distribution of predicted cysteine oxidation rates across the seven main enzyme classes (EC 1 oxidoreductases, EC 2 transferases, EC 3 hydrolases, EC 4 lyases, EC 5 isomerases, EC 6 ligases and EC 7 translocases) was analysed. Hydrolases are significantly more likely to be predicted as oxidatively modified compared with all other enzyme classes. This supports the hypothesis of radio­lytic generation of ROS which oxidatively modify the cysteine, as hydrolases are poised to activate water for nucleophilic attack of their substrates (Rao *et al.*, 1998 ▸; Cui *et al.*, 2014 ▸; He *et al.*, 2020 ▸; Luang *et al.*, 2025 ▸). We further investigated whether PDB entries which contain at least one EC number annotated in the M-CSA database (Ribeiro *et al.*, 2018 ▸) as utilizing a cysteine in their active site are more likely to be predicted as damaged. Our results suggest that enzymes with an active-site cysteine are ∼61% more likely to be predicted as damaged compared with others. At the time of writing, there are 6914 currently active enzyme classification numbers according to the ExplorEnz database (McDonald *et al.*, 2009 ▸), showing that our analysis is limited, as there are only 3857 in the cysteine-containing PDB entries within the dataset; although 6429 cysteine-containing structures are unannotated with EC information. Furthermore, only 837 EC numbers are represented in the M-CSA database. Therefore, whilst the analysis here attempts to correlate enzyme function with predicting oxidatively damaged cysteines, this may only apply to data accessible within the PDB and might not extend to EC numbers which are not represented in the manually curated M-CSA database.

It is worth noting that our workflow only reports whether a cysteine has positive difference density in accordance with oxidative damage to a cysteine and does not attempt to fit or refine these modifications. Therefore, false positives are present and can be caused by modelling the cysteine as unoccupied, unmodelled ligands or by altogether poorly modelled cysteines.

This work aimed to highlight that cysteine oxidation can occur during X-ray data collection. Therefore, if evaluating the oxidation state of cysteine is important for understanding protein function, careful attention should be paid to the conditions under which the data are collected. Furthermore, structures should be carefully reviewed before deposition to ensure they are as accurate as possible, in an age where deep-mining and machine-learning models are being increasingly used to predict pharmaceutically relevant drug targets.

## Supplementary Material

Supplementary Tables. DOI: 10.1107/S2059798326003943/gm5124sup1.pdf

## Data Availability

All of the code used in the dataset construction is open-source and is available on GitHub at https://github.com/Samuel-Foster62/Cys_Ox_Analysis. The final parsed flat files are also available at this location, enabling looking up of each structure from the four-digit accession code in the Protein Data Bank repository (https://www.wwpdb.org/)

## Footnotes

1 PDB entries 1j0x, 1obf, 2g82, 2vyn, 2vyv, 5c7i, 5c7l, 5c7o, 5t73, 5ur0, 6qun, 6utn, 6ynd, 6ynf, 6ynh, 7jh0, 7u4s, 7xzg, 7zq4, 8de5 and 8p5f.
