# Supplementary material for: It started off as a Cys, how did it end up like this? Identifying the extent of unmodelled oxidatively modified cysteines within the Protein Data Bank
Source: Acta Crystallogr D Struct Biol. 2026 Jul 1;82(Pt 7):700–14. doi: 10.1107/S2059798326003943 (PMC13317685; doi:10.1107/S2059798326003943)
Supplement: Supplementary file 1 [file d-82-00700-sup1.pdf]

STRUCTURAL
BIOLOGY

Volume 82 (2026)

Supporting information for article:

It started off as a Cys, how did it end up like this? Identifying the extent of unmodelled oxidatively modified cysteines within the Protein Data Bank

Samuel P. Foster, Anna J. Warren, C. Alistair Siebert, Jaswir Basran and Peter C. E. Moody

Table S1 Two-tailed Kolmogorov-Smirnov test statistic (Massey, 1951) and *P*-values between undamaged and damaged cysteines, for the 10 parameters used in the GAPDH classification dataset

Parameter	Kolmogorov-Smirnov Statistic, D	*P*-value
RSZD	0.945	<0.0001
RSCC	0.478	<0.0001
Number of peaks	0.782	<0.0001
RMSD	0.784	<0.0001
Minimum distance to Cys SG	0.786	<0.0001
Maximum distance to Cys SG	0.788	<0.0001
$B_{net\text{-}}$percentile	0.120	=0.0204
$\sigma(B_{Damage})$	0.159	=0.0002
Temperature	0.145	=0.0010
Resolution	0.076	=0.2367

Table S2 Expected sulphur-oxygen bond distances for oxidised cysteines extracted from ideal ligand coordinates available in the PDB.

Residue name	Modification		PDB ligand code	SG-O order	SG-O distance (Å)
Cysteine Sulfenic Acid	Sulfenate	RSO^-	CSO	Single	1.522
Cysteine Sulphoxide	Sulphoxide	RSO	CSX	Double	1.495
Cysteine Sulfinic Acid	Sulfinate	RSO_2^-	CSD	Single	1.522
				Double	1.421
Cysteine Sulfonic Acid	Sulfonate	RSO_3^-	OCS	Single	1.521
				Double	1.421
				Double	1.421
Cysteine S sulfonic acid	Sulfonate	$RSSO3^-$	CSU	Single	1.631
				Double	1.450
				Double	1.450

Table S3 Performance metrics for the classifiers ability to correctly detect oxidatively damaged cysteines, evaluated using the validation dataset. An RSZD ≥ 3σ and a minimum distance to difference map peak between 1.00 and 2.50Å were set as the thresholds using the GAPDH dataset. TN, TP, FN, FP are true negative, true positive, false negative and false positive respectively, where negative refers to undamaged and positive refers to oxidatively damaged.

Performance metric	Equation	Value
Accuracy	$\frac{TN + TP}{TN + TP + FN + FP}$	0.962
Sensitivity	$\frac{TP}{TP + FN}$	0.679
Specificity	$1 - False\ Positive\ Rate$	0.965
Precision (Positive Predictive Value)	$\frac{TP}{TP + FP}$	0.207
Negative Predictive Value (NPV)	$1 - \frac{FN}{FN + TN}$	0.996
F1 score	$2 \times \frac{Sensitivity \times Precision}{Sensitivity + Precision}$	0.317
False Positive Rate	$\frac{FP}{FP + TN}$	0.035
False Negative Rate	$1 - Sensitivity$	0.321
Diagnostic Odds Ratio (DOR)	$\frac{TP/FN}{FP/TN}$	58.9
95% Confidence interval DOR	$\ln(DOR) \pm 1.96 \times \sqrt{\frac{1}{TP} + \frac{1}{FN} + \frac{1}{FP} + \frac{1}{TN}}$	25.8 – 134.6
Matthew's Correlation Coefficient (MCC)	$\frac{TP \times TN - FP \times FN}{\sqrt{(TP + FP)(TP + FN)(TN + FP)(TN + FN)}}$	0.361

Table S4 2×2 table comparing predicted PDB-level damage (flagged with damage if ≥1 cysteine) with whether the PDB contains at least one EC number from the M-CSA catalytic cysteine set ($EC_{M\text{-}CSA}$)

	Predicted damaged	Predicted undamaged	Total
PDB contains EC in $EC_{M\text{-}CSA}$	12,678	32,300	44,978
PDB does not contain EC in $EC_{M\text{-}CSA}$	18,435	54,802	73,237
Total	31,113	87,102	118,215

Reference

Massey, F. J. (1951). *J. Am. Stat. Assoc.* **46**, 68–78.
